# Multi‐site sequencing supports dual‐track evolution and identifies candidate metastasis‐associated factors in gastric cancer ovarian metastasis

**DOI:** 10.1002/ctm2.70847

**Published:** 2026-09-21

**Authors:** Peiyu Zhu, Xiaofang Xing, Ke Ji, Lingqian Wang, Zhongwu Li, Shuqin Jia, Longtao Huangfu, Xiaomei Li, Biao Fan, Zhaode Bu, Jiafu Ji

**Affiliations:** ^1^ Key Laboratory of Carcinogenesis and Translational Research (Ministry of Education) Gastrointestinal Cancer Center Peking University Cancer Hospital & Institute Beijing China; ^2^ State Key Laboratory of Holistic Integrative Management of Gastrointestinal Cancers Beijing Key Laboratory of Carcinogenesis and Translational Research Division of Gastrointestinal Cancer Translational Research Laboratory Peking University Cancer Hospital & Institute Beijing China; ^3^ Key Laboratory of Carcinogenesis and Translational Research (Ministry of Education) Department of Pathology Peking University Cancer Hospital & Institute Beijing China; ^4^ State Key Laboratory of Holistic Integrative Management of Gastrointestinal Cancers Beijing Key Laboratory of Carcinogenesis and Translational Research Center for Molecular Diagnostics Peking University Cancer Hospital & Institute Beijing China; ^5^ State Key Laboratory of Holistic Integrative Management of Gastrointestinal Cancers Gastrointestinal Cancer Center Peking University Cancer Hospital & Institute Beijing China

**Keywords:** clonal evolutionary trajectory, gastric cancer ovarian metastasis (GCOM), multi‐site sequencing, *SCAF4*

## Abstract

**Background:**

Gastric cancer ovarian metastasis (GCOM) is an aggressive clinical entity whose evolutionary dissemination routes and molecular features remain incompletely defined.

**Methods:**

We performed whole‐exome sequencing and patient‐level phylogenetic reconstruction of 81 spatially distinct tumour specimens from 17 patients with GCOM.

**Results:**

Ovarian metastases showed a higher tumour mutational burden than matched primary tumours and substantial inter‐lesional genomic heterogeneity. Phylogenetic reconstruction supported two major evolutionary routes of ovarian dissemination: lymph node‐dependent evolution, in which ovarian metastases were phylogenetically associated with lymph node‐related lineages, and lymph node‐independent evolution, in which ovarian and lymph node metastases diverged along separate branches. Putative driver‐gene alterations were relatively enriched in the shared/truncal mutational fraction, and *SCAF4* emerged as an understudied candidate metastasis‐associated factor. In GC cells, *SCAF4* depletion was accompanied by widespread alterations in RNA splicing and interferon‐related transcriptional programs and enhanced proliferative, migratory, invasive, and clonogenic phenotypes.

**Conclusions:**

These findings support a dual‐route evolutionary framework for GCOM and nominate *SCAF4* as a candidate metastasis‐associated factor, providing a genomic basis for understanding the biological heterogeneity of ovarian dissemination in GC.

**Highlights:**

- A multi‐site genomic sequencing and phylogenetic reconstruction in matched gastric primaries and ovarian metastases was performed.
- Uncovered two distinct evolutionary routes for gastric cancer ovarian metastasis: lymph node‐dependent and lymph node‐independent evolutionary patterns.
- Genomic and functional analyses nominate *SCAF4* as a candidate metastasis‐associated factor.

## Graphical abstract

- Multi‐site whole‐exome sequencing and phylogenetic reconstruction characterise gastric cancer ovarian metastasis.
- Lymph node‐dependent and lymph node‐independent evolutionary patterns coexist across patients.
- *SCAF4* is nominated as a candidate metastasis‐associated factor supported by genomic and loss‐of‐function evidence.

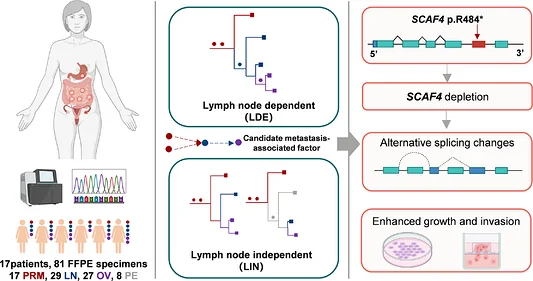

## 1 INTRODUCTION

Gastric cancer (GC) metastasis to the ovaries (GCOM) constitutes a uniquely lethal disease endpoint, characterised by an aggressive clinical course and marked therapeutic resistance.^1^, ^2^, ^3^, ^4^, ^5^ For over a century, its underlying biology has been a subject of debate, with competing hypotheses—sequential lymphatic spread, direct transcoelomic seeding, and hematogenous dissemination—impeding clinical progress.^6^, ^7^ This long‐standing uncertainty is not merely an academic question; it has created a clinical stalemate, precluding the development of rational biomarkers to stratify high‐risk patients or pathway‐specific therapies to intercept the metastatic cascade. Consequently, GCOM remains a devastating condition managed with a one‐size‐fits‐all approach that has yielded little improvement over decades.

This knowledge gap is particularly striking when contrasted with the conceptual revolutions that have reshaped our understanding of metastasis in other solid tumours. The classical model of linear, lymph node‐mediated progression has been refined by high‐resolution genomic and phylogenetic tracing, which has uncovered a complex landscape of dissemination routes in cancers such as melanoma, colorectal cancer, and breast cancer.^8^, ^9^, ^10^ These studies have provided evidence of direct hematogenous dissemination bypassing lymph nodes, parallel seeding of multiple metastatic sites, and even metastasis‐to‐metastasis spread.^11^, ^12^, ^13^, ^14^, ^15^ Yet, GCOM has remained largely unexplored using these modern approaches, leaving a critical question unanswered: does it follow a single canonical path, or does it leverage the diverse evolutionary strategies now recognised in other cancers?

To address this gap, we performed multi‐site whole‐exome sequencing of 81 tumour specimens from 17 patients with GCOM, integrating primary gastric tumours with matched lymph node, ovarian, and, when available, peritoneal metastases. Patient‐specific phylogenetic reconstruction supported two major evolutionary patterns of ovarian dissemination, consistent with lymph node‐dependent evolution (LDE) and lymph node‐independent evolution (LIN). We further investigated genomic alterations associated with metastatic evolution and identified *SCAF4* as a candidate metastasis‐associated factor, supported by transcriptomic and functional analyses in GC cells. Together, these findings provide a genomic framework for understanding the evolutionary heterogeneity of GCOM.

## 2 MATERIALS AND METHODS

### 2.1 Tissue samples from GC patients

We included 17 patients with GCOM who were treated at Beijing Cancer Hospital between November 2014 and March 2020 and underwent gastrectomy together with concurrent or staged oophorectomy. In total, 81 formalin‐fixed paraffin‐embedded (FFPE) tumour specimens were analysed, comprising 17 primary gastric tumours and 64 matched metastatic lesions, including 29 metastatic lymph nodes, 27 ovarian metastases and eight peritoneal metastases. Tumour‐containing regions of metastatic specimens were identified and isolated by microdissection before downstream analyses. Ovarian metastasis present at the time of initial diagnosis or staging was classified as synchronous, whereas metastasis first detected during subsequent follow‐up was classified as asynchronous. Primary tumours, lymph node metastases, ovarian metastases, and peritoneal metastases were obtained through gastrectomy, lymphadenectomy, oophorectomy and surgical resection of peritoneal nodules, respectively. Clinicopathological characteristics and treatment information are summarised in Table S1. This retrospective study was approved by the Ethics Committee of Beijing Cancer Hospital (approval No. 2024YJZ56). Written informed consent for the collection, storage, and scientific use of tumour specimens had been obtained from all patients before surgery.

### 2.2 Whole‐exome sequencing

FFPE specimens were reviewed by experienced gastrointestinal pathologists, and tumour‐rich regions were identified on H&E‐stained sections. Serial 5‐µm‐thick sections were subjected to laser microdissection using an MMI CellCut Plus system. Multiple serial sections were processed as needed to obtain sufficient tumour DNA for library preparation and whole‐exome sequencing (WES).

Genomic DNA was isolated from FFPE tumour tissue using the QIAamp DNA FFPE Tissue Kit, whereas DNA from matched peripheral blood was purified using the QIAamp DNA Blood Midi Kit (Qiagen, Hilden, Germany). DNA integrity was evaluated by 1% agarose gel electrophoresis, and DNA concentration was determined using the Qubit DNA Assay Kit on a Qubit 2.0 fluorometer (Invitrogen, USA). For each specimen, 0.6 µg of genomic DNA was used for library construction. Exome enrichment was performed with the SureSelect Human All Exon V6 kit (Agilent Technologies), followed by sequencing on an Illumina HiSeq X Ten platform (Illumina, San Diego, CA). Sample‐level sequencing quality metrics, including raw and clean read counts, Q30, GC content, mapping rate, and duplication rate, are provided in Table S2.

Somatic single‐nucleotide variants (SNVs) and indels were identified using GATK Mutect2 v4.3.0.0 against the hg19 reference genome, incorporating a panel of normals and the gnomAD germline resource. Read‐orientation artefacts were modelled with LearnReadOrientationModel, whereas GetPileupSummaries and CalculateContamination were used to estimate sample contamination. Variant filtering was performed with FilterMutectCalls, and only PASS calls were retained. Functional and database annotations were subsequently added using ANNOVAR. To increase the robustness of somatic mutation detection, Mutect2 PASS variants were included in the final consensus set only when the same genomic allele was independently supported by either VarScan or Strelka. Variants with an East Asian population allele frequency >1% in available population databases were removed. Candidate variants were additionally required to have sequencing depths >10× in both tumour and matched‐normal specimens, at least three alternative‐allele‐supporting reads in the tumour, and no more than one alternative read in the matched normal.

### 2.3 Mutational signature analysis

Somatic SNVs from the final consensus mutation set were classified into the conventional 96 trinucleotide substitution contexts using the hg19 reference genome. De**single‐base substitution (SBS)** signatures were extracted by non‐negative matrix factorisation using maftools (v2.14.0) and the NMF package (v0.28). The signature rank was evaluated before extraction of a two‐signature solution. The resulting spectra were compared with COSMIC SBS reference signatures using cosine similarity, and sample‐level signature contributions were obtained from the NMF decomposition.

### 2.4 Driver‐gene annotation and pathway analysis

The 299 consensus cancer driver genes reported by Bailey et al.^8^ were used as the reference driver‐gene set. Genes overlapping with Tier 1 of the COSMIC Cancer Gene Census were classified as established cancer driver genes, whereas the remaining genes in the Bailey consensus set were classified as candidate driver genes. Protein‐altering somatic variants affecting these genes were termed putative driver‐gene alterations. Functional/coding somatic variants were grouped according to lesion site (primary tumour, lymph node metastasis, peritoneal metastasis and OM). Within each group, mutated genes were pooled and deduplicated, and the resulting site‐specific gene lists were independently subjected to KEGG pathway over‐representation analysis using DAVID.

### 2.5 Publicly available TCGA data

For the pan‐cancer TMB comparison, mutation burden across 33 TCGA cancer cohorts was obtained using the tcgaCompare function implemented in maftools, which uses somatic mutation data from the TCGA MC3 project. For the mutation‐frequency comparison, an analysis‐specific Asian subset of TCGA‐STAD was defined using demographic metadata from the recount3 TCGA resource. Cases annotated as Asian with primary‐tumour samples were retained, with one aliquot selected per case. Of 87 eligible cases, 85 had evaluable open‐access masked somatic mutation MAF files from the NCI Genomic Data Commons and were included in mutation‐frequency calculations. Protein‐altering variants included missense, nonsense, nonstop, frameshift, in‐frame indel, splice‐site, and translation‐start‐site mutations. Each case was counted once per gene, and mutation frequency was calculated as the proportion of mutated cases among the 85 evaluable tumours.

**FIGURE 1:**
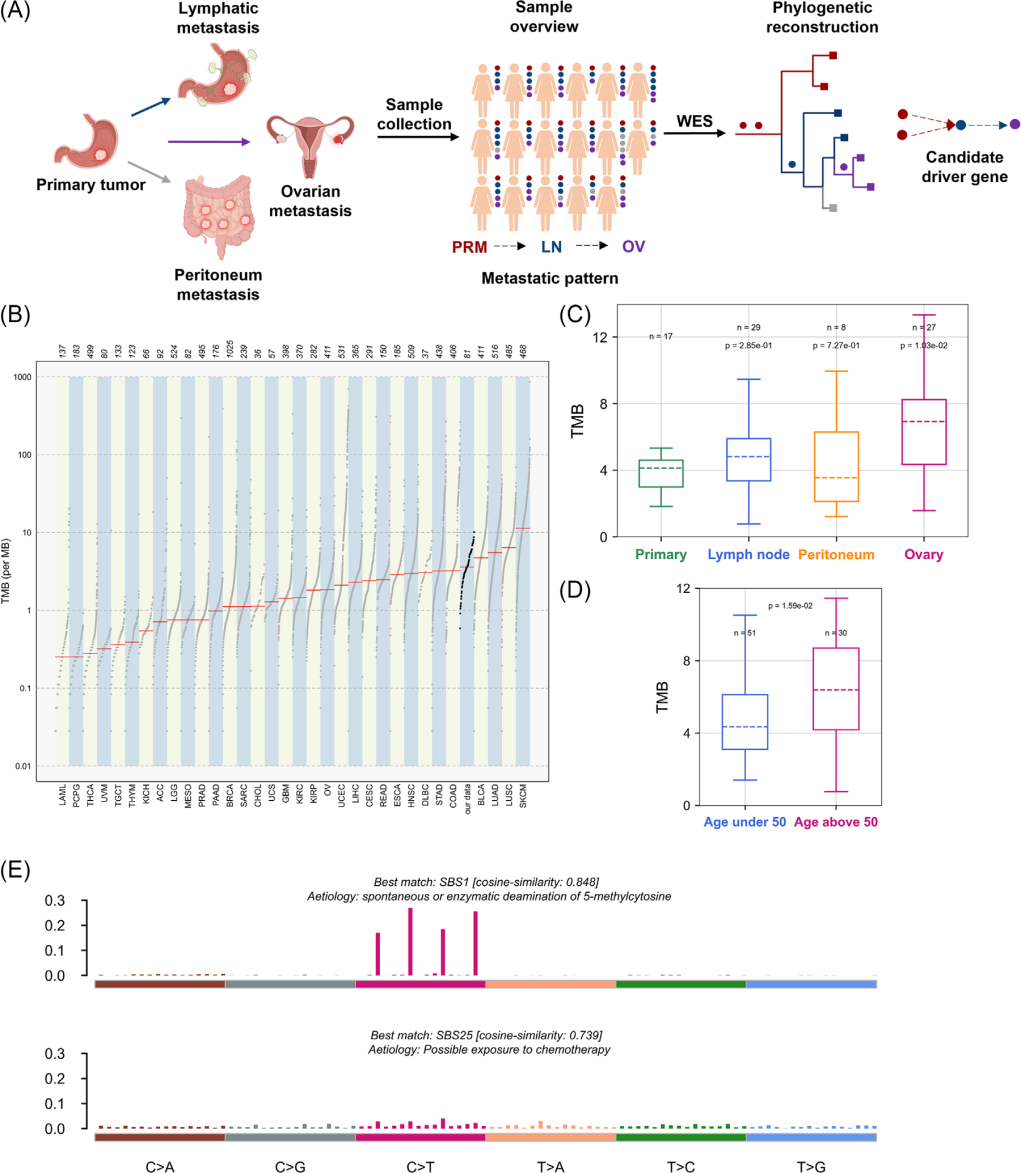
Mutational features of primary and metastatic foci of gastric cancer. (A) Study design schematic showing the collection of primary tumours (PRM), lymph node metastases (LN), ovarian metastases (OV) and peritoneal metastases (PE) for whole‐exome sequencing (WES), followed by patient‐level phylogenetic reconstruction and functional validation of candidate genes. (B) Comparison of tumour mutational burden (TMB) between the gastric cancer ovarian metastasis (GCOM) cohort and 33 TCGA cancer types. (C) Comparison of TMB across anatomical sites. (D) Comparison of TMB between patients aged <50 and ≥50 years. (E) Mutational profiles of SBS1 and SBS25.

### 2.6 Phylogenetic tree construction

High‐confidence somatic SNVs from all available tumour sites within each patient were used to reconstruct phylogenetic trees with Treeomics v1.9.2. Treeomics applies a Bayesian framework to infer mutation presence or absence while accounting for sequencing uncertainty and variation in sample tumour cellularity; a minimum of three variant‐supporting reads was required. Pairwise genetic distances were calculated within each patient. Cases were classified as LDE when OV lesions clustered within or most closely with an LN‐related lineage, and as LIN when OV and LN lesions occupied separate branches from an earlier common ancestor.

### 2.7 Immunohistochemistry

FFPE tissue sections (5 µm) were mounted on poly‐L‐lysine‐coated slides. Endogenous peroxidase activity was quenched with 3% hydrogen peroxide. Antigen retrieval was performed by pressure cooking in 10 mmol/L EDTA buffer (pH 8.0) for 3 min. Sections containing gastric primary tumours and ovarian metastatic lesions were then incubated with the indicated primary antibodies at 4°C overnight, followed by incubation with a horseradish peroxidase‐conjugated secondary antibody at room temperature for 30 min. Signals were developed using DAB, and nuclei were counterstained with Mayer's haematoxylin.

### 2.8 Cell lines

Human GC cell lines SNU484 and AGS were obtained from the Korean Cell Line Bank (KCLB, Seoul, Republic of Korea) and the American Type Culture Collection (ATCC, Manassas, VA, USA), respectively. Cells were maintained in RPMI‐1640 medium (GIBCO BRL, Carlsbad, CA) supplemented with 10% (v/v) foetal calf serum (FCS; GIBCO) and antibiotics at 37°C in a humidified atmosphere containing 5% CO_2_.

### 2.9 Lentiviral SCAF4 knockdown and establishment of stable cell lines

Lentiviral vectors carrying shRNAs targeting human SCAF4 were provided by GeneMediTech (Shanghai, China). The knockdown sequences were shRNA1847: CCCGATCTCGATCTCAAGAAA; shRNA646: CGTCATCAGTTTGAACTGAT; and shRNA1109: AGGCTATCACAGCTCAGTTAA. Lentiviral particles were used to simultaneously infect SNU484 and AGS cells according to the manufacturer's protocol. A non‐targeting shRNA (NT) was included as the control. Stable cell populations were obtained by selection with puromycin at 1.0 µg/mL.

### 2.10 RNA sequencing and transcriptomic analyses

RNA sequencing was performed in control and SCAF4‐knockdown AGS and SNU484 cells, with three biological replicates included for each condition. Alternative splicing was quantified using rMATS, and events meeting a false discovery rate (FDR) ≤0.05 and |ΔPSI| ≥0.10 were retained. Differential gene expression was analysed using edgeR with a statistical model incorporating both cell line and knockdown status. For Hallmark gene‐set enrichment analysis, genes were ranked according to the cell‐line‐adjusted edgeR statistic, and Benjamini‐Hochberg‐adjusted FDR values were reported. Sample‐level single‐sample gene‐set enrichment scores were additionally calculated for five immune‐related programs. Differences between knockdown and control groups were assessed using 400 exact within‐cell‐line permutations followed by Benjamini‐Hochberg correction.

### 2.11 Colony formation assays

For colony‐formation assays, GC cells were plated in six‐well plates at a density of 600 cells per well and maintained for 14 days at 37°C in 5% CO_2_. Colonies were subsequently rinsed twice with phosphate‐buffered saline, fixed with 75% ethanol for 15 min at 21°C, and stained with 0.1% crystal violet. After excess dye was removed, the plates were air‐dried and photographed.

### 2.12 Wound‐healing assay

GC cells were cultured in six‐well plates until a confluent monolayer was obtained. A linear wound was then generated using a sterile 200‐µL pipette tip, and detached cells were removed by washing with phosphate‐buffered saline. Images of the wound area were acquired at 0, 24 and 48 h using an inverted phase‐contrast microscope at 200× magnification. Cell migration was quantified on the basis of the mean migration distance.

### 2.13 Transwell and invasion assays

Cell invasive capacity was evaluated using Transwell inserts equipped with 8‐µm‐pore polycarbonate membranes. GC cells were suspended in serum‐free medium and added to the upper chamber at a density of 10^5^ cells/mL, while medium containing 10% FCS was placed in the lower chamber. After incubation for 48 h at 37°C, cells that had traversed the membrane and reached its lower surface were stained and counted.

### 2.14 Statistical analysis

Comparisons of categorical variables were performed using the chi‐squared test or Fisher's exact test, as appropriate. Continuous variables were evaluated using two‐sided Student's t tests, Mann‐Whitney *U* tests, or Wilcoxon signed‐rank tests according to the study design and distribution of the data. Cell‐based experimental data were analysed using two‐sided Student's *t* tests. Prespecified patient subgroup analyses were considered exploratory, and nominal two‐sided *p*‐values are reported.

## 3 RESULTS

### 3.1 Genomic landscape of primary and metastatic foci of GC

To delineate the genomic landscape and evolutionary patterns of GCOM, we performed WES on a unique cohort of 81 spatially distinct tumour samples from 17 patients (Figure 1A). This comprehensive dataset included primary gastric tumours (PRM, *n* = 17), matched lymph node metastases (LN, *n* = 29), peritoneal metastases (PE, *n* = 8), and ovarian metastases (OV, *n* = 27). Initial characterisation of the mutational landscape revealed that missense mutations were the most prevalent variant type across all lesion sites (Figure S1A).

First, we evaluated the tumour mutational burden (TMB) across our cohort. Comparison with 33 common tumour types from the Cancer Genome Atlas (TCGA) database revealed that GCs in our cohort exhibited a generally high TMB, comparable to that in TCGA GC samples (Figure 1B). Further intra‐patient analysis demonstrated that while TMB levels were largely consistent among primary gastric tumours, lymph node metastases, and peritoneal metastases, ovarian metastases displayed significantly higher TMB than primary tumours (Figure 1C). LN TMB was not significantly associated with whether the lesion was classified as sentinel or secondary (Figure S1B), and samples from patients aged ≥50 years showed higher TMB than those from younger patients (Figure 1D). The higher TMB observed in OV may reflect the combined effects of elapsed time, treatment exposure, clonal selection, and site‐specific tumour evolution.

Crucially, we investigated whether the timing of metastatic presentation accounted for this increased mutational complexity. While no significant TMB differences were found between synchronous and asynchronous samples in primary tumours or peritoneal sites, asynchronous metastases in both lymph nodes and ovaries exhibited significantly higher TMB compared to their synchronous counterparts (Figure S1D). However, many asynchronous specimens were collected after systemic treatment. Accordingly, these differences may reflect the combined effects of metastatic site, elapsed time, intrinsic tumour evolution, spatial heterogeneity, treatment‐induced mutagenesis and treatment‐mediated clonal selection, rather than untreated natural evolution alone.

To investigate the underlying mutational processes, we conducted mutational signature analysis. We identified predominant signatures consistent with spontaneous deamination of 5‐methylcytosine (SBS1) and those potentially associated with chemotherapy exposure (SBS25) (Figure 1E), with a global dominance of C > T transitions and a high transition‐to‐transversion (Ti/Tv) ratio (Figure S1C), typical of gastric adenocarcinoma profiles. These findings provide initial insights into the genomic features driving GCOM progression and suggest that ovarian metastases may experience distinct evolutionary pressures, potentially accumulating additional mutations.

### 3.2 Shared driver mutations and convergent pathway activation define GCOM evolution

**FIGURE 2:**
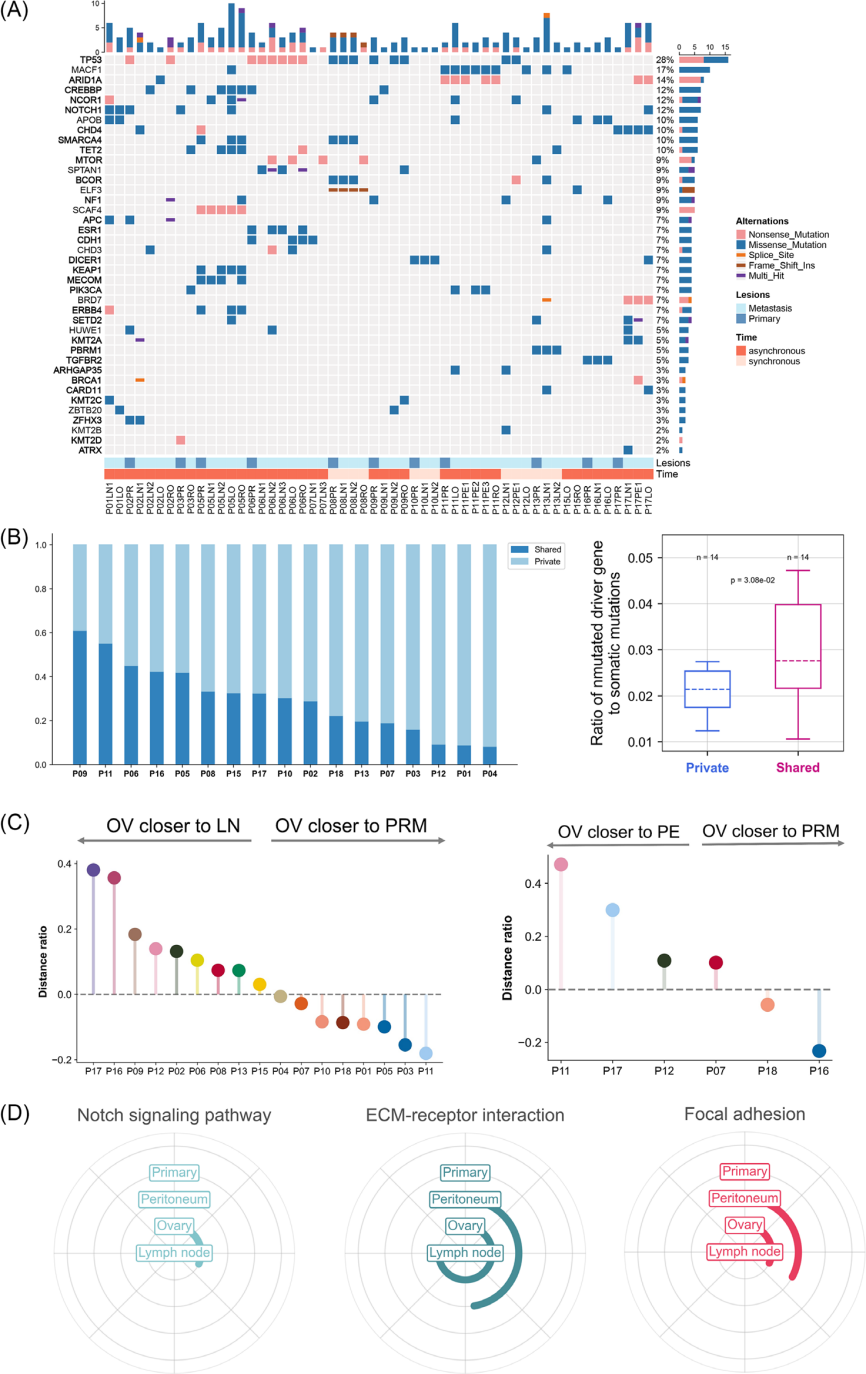
Shared driver mutations and convergent pathway activation define gastric cancer ovarian metastasis (GCOM) evolution. (A) The SNVs of the candidate genes with frequencies higher than 2% are displayed. Each column corresponds to one sample. The top graph shows mutation numbers. The right graph indicates the relative frequencies of a specific gene. Genes overlapping between the Bailey consensus cancer driver‐gene set and Tier 1 of the COSMIC Cancer Gene Census are shown in bold and were classified as established cancer driver genes; the remaining genes from the Bailey consensus set are shown in regular font and were classified as candidate driver genes. (B) Patient‐level proportions of shared and private mutations and the proportion of putative driver‐gene alterations within each fraction (evaluable patients, *n* = 14; two‐sided paired Wilcoxon signed‐rank test; exact *p*‐value shown). (C) Genetic distance between primary and metastatic foci. (D) KEGG pathway enrichment of primary and metastatic foci. Clockwise arc length represents −log10(*P*), whereas arc colour distinguishes pathways.

To elucidate the clonal architecture and identify candidate driver events in GCOM, we first analysed the landscape of somatic mutations across all 17 patients (Figure 2A). Our analysis confirmed a high prevalence of mutations in well‐established GC driver genes, including *TP53*, *CDH1*, *CREBBP* and *ARID1A*. Within individual patients, a subset of these alterations was shared across the primary tumour and metastatic specimens, consistent with acquisition before or early during dissemination.

We next systematically quantified the contribution of shared (truncal) versus private (branch‐specific) mutations to the driver gene pool. A clear pattern emerged: the ratio of putative driver mutations to all somatic mutations was significantly higher in the shared clonal fraction than in the private fraction (Figure 2B). This observation supports consideration of early shared events when prioritising candidate genes, but does not imply that every truncal alteration is a metastasis driver.

Building on this clonal framework, we directly investigated the evolutionary routes of dissemination by calculating genetic distances between distinct metastatic sites. This analysis revealed two distinct patterns. In a majority of patients, OV were genetically closer to LN than to PRM (Figure 2C, left panel, Figure S2A). Among patients with available PE specimens, the relationship was heterogeneous, with OV being genetically closer to either PE or PRM in different cases (Figure 2C, right panel, Figure S2B).

Finally, to uncover the functional programs required for ovarian colonisation, we performed pathway enrichment analysis. This analysis showed site‐associated differences in pathways related to cell migration and adhesion, with higher enrichment scores observed in ovarian metastases. Notably, programs such as ECM‐receptor interaction and focal adhesion were significantly enriched in ovarian tumours but not in their matched primary, lymph node, or peritoneal counterparts (Figure 2D and Figure S3). These site‐associated pathway patterns are consistent with a potential role for cell‐matrix interactions in ovarian colonisation.

### 3.3 Somatic alterations in GCOM converge on pathways promoting metastasis and epigenetic dysregulation

**FIGURE 3:**
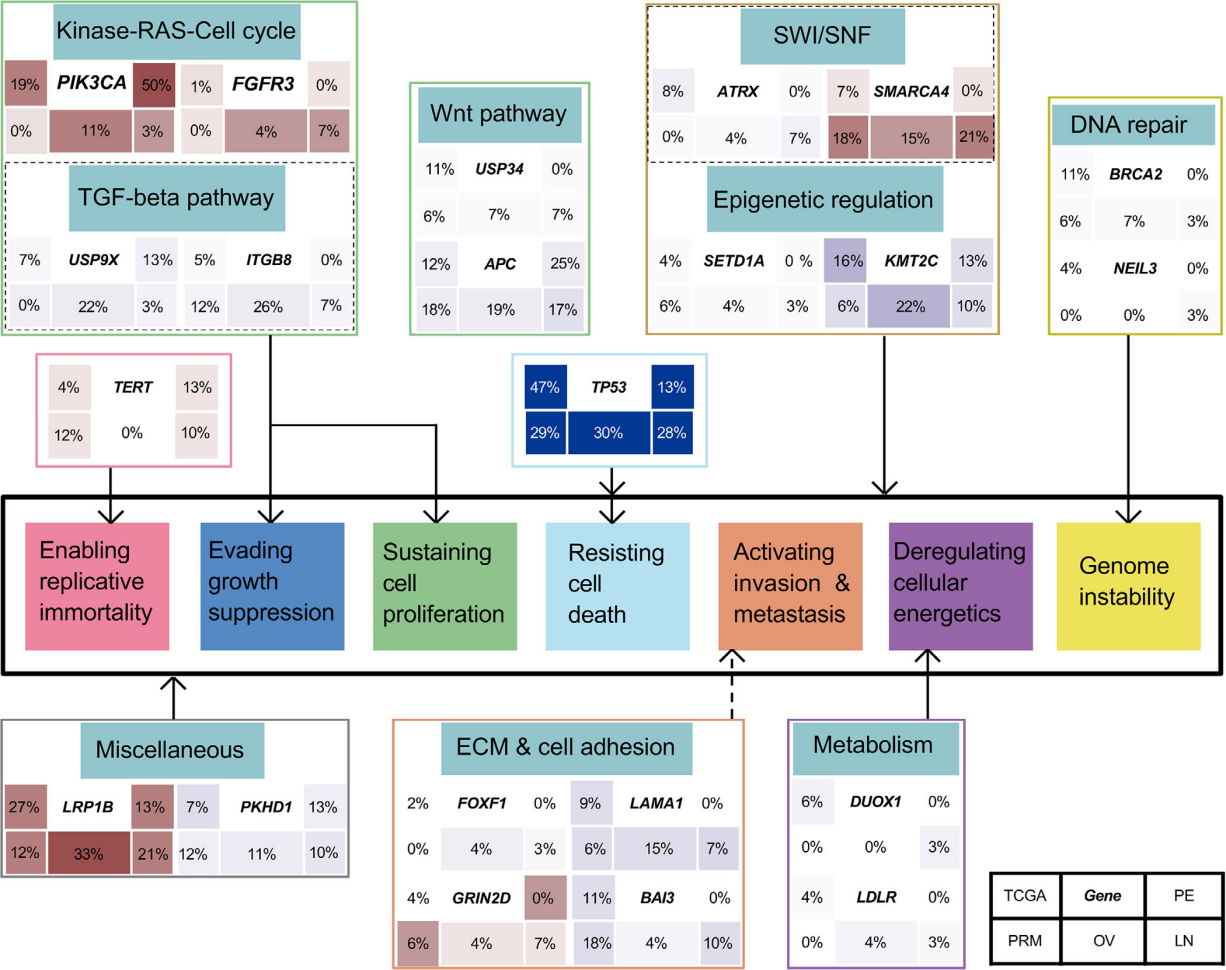
Protein‐altering genomic alterations mapped to cancer‐related pathways and hallmarks across primary tumours (PRM) (*n* = 17), ovarian metastases (OV) (*n* = 27), lymph node metastases (LN) (*n* = 29) and peritoneal metastases (PE) (*n* = 8) specimens, with TCGA‐STAD Asian primary tumours (*n* = 85) shown as a descriptive external comparator. Percentages indicate the proportion of specimens harbouring an alteration in the indicated gene. The inset defines the six‐cell layout used for each gene: TCGA, gene label and PE in the upper row; PRM, OV and LN in the lower row.

To elucidate the functional consequences of the observed genomic alterations, we mapped all non‐synonymous mutations to established cancer‐related pathways and the “Hallmarks of Cancer” (Figure 3). This analysis revealed that while core oncogenic pathways were consistently altered, mutations in genes governing cell adhesion and epigenetic state were distinctly enriched, particularly in metastatic lesions.

As expected, fundamental cancer hallmarks were driven by high‐frequency mutations in well‐established driver genes. The “Resisting cell death” and “Sustaining cell proliferation” hallmarks were predominantly affected by recurrent mutations in TP53, which were present across all tumour sites, including PRM (29%), OV (30%), LN (28%) and PE (13%). Similarly, pathways such as the Kinase‐RAS‐Cell Cycle axis were recurrently altered via mutations in genes like PIK3CA.

More strikingly, the analysis highlighted a specific mutational signature associated with the “Activating invasion & metastasis” hallmark. We observed significant enrichment of somatic mutations in genes related to extracellular matrix (ECM) and cell adhesion, especially in ovarian metastases. Notably, the laminin subunit gene LAMA1 was mutated in 15% of ovarian samples, representing an increase compared to other sites. Similarly, LRP1B, a receptor involved in cell adhesion and signalling, harboured mutations in 33% of ovarian and 13% of peritoneal metastases. These genomic patterns are consistent with a potential involvement of cell‐matrix interactions in OM.

Furthermore, we identified frequent alterations in genes linked to epigenetic regulation. Key chromatin remodelling genes, including KMT2C and members of the SWI/SNF complex such as SMARCA4, were mutated in 6%‐22% of metastatic samples. These findings suggest that epigenetic dysregulation may contribute to the molecular heterogeneity of GCOM.

In summary, beyond canonical alterations in tumour suppressors like TP53, the genomic landscape of GCOM is uniquely characterised by the selection of mutations in genes controlling cell‐matrix adhesion and epigenetic programming. These alterations provide a functional basis for its aggressive metastatic phenotype.

### 3.4 Complex genetic evolution showed the heterogeneity of OM from GC

**FIGURE 4:**
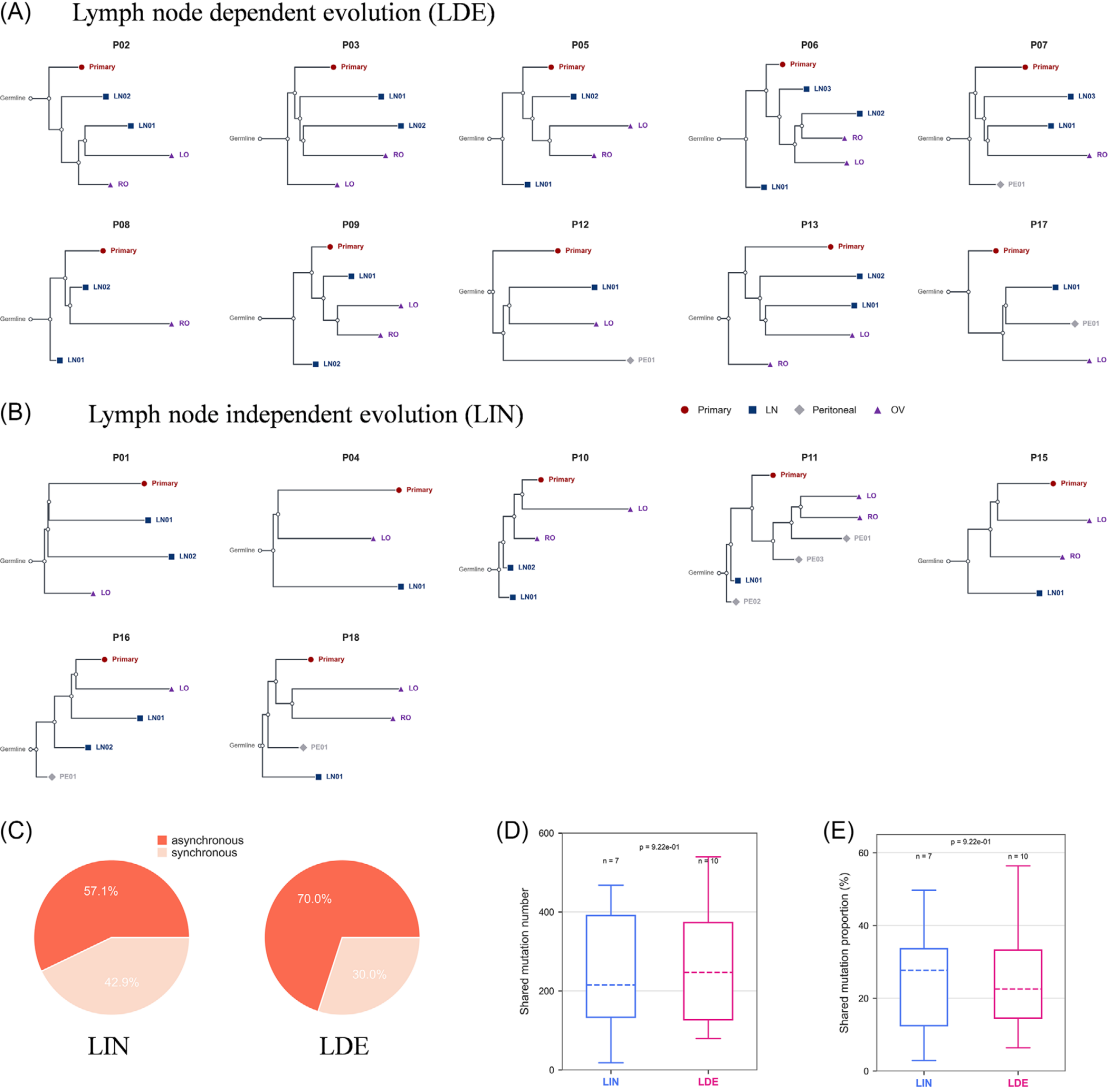
Phylogenetic reconstruction of metastatic spread in GCOM. (A) Treeomics phylogenies for the 10 cases classified as lymph node‐dependent evolution (LDE). (B) Treeomics phylogenies for the seven cases classified as lymph node‐independent evolution (LIN). Symbols denote PRM (red circles), LN (blue squares), PE (grey diamonds) and OV (purple triangles); LO and RO indicate left and right ovarian metastases, respectively. (C) Proportions of synchronous metastasis and asynchronous metastasis in two models. (D, E) Shared mutation number and shared mutation proportion in LIN (*n* = 7) and LDE (*n* = 10); exact *p*‐values are shown (two‐sided Mann‐Whitney U tests).

To further analyse the genomic evolutionary trajectories of GCOM, we used Treeomics to reconstruct phylogenetic trees for metastatic lesions across different cases. Based on these trees, the evolutionary pathway of GCOM is distinguished into two models: LDE and LIN. As shown in Figure 4A and Table S3, ten patients showed patterns consistent with LDE, in which OV clustered within or most closely with an LN‐related lineage. Seven patients showed LIN, in which OV and LN occupied separate branches from an earlier common ancestor. (Figure 4B). These results support two models of GCOM: the LDE model, wherein OM occurs via initial seeding from the primary tumour to lymph nodes followed by subsequent metastasis to ovaries; and the LIN model, wherein ovarian and lymph node metastases occupied separate phylogenetic branches, consistent with ovarian dissemination occurring independently of the sampled lymph node lineage.

We next investigated whether these evolutionary routes correlated with clinical presentation and mutational characteristics. Notably, the LDE route was predominantly associated with asynchronous metastasis, accounting for 70% of cases, whereas the LIN route showed a more balanced distribution between synchronous and asynchronous presentations (57.1% vs. 42.9%; Figure 4C). This suggests that the LDE pathway may represent a more protracted evolutionary process requiring cumulative seeding events.

We next compared the extent of mutational sharing between the two evolutionary groups. Neither the number nor the proportion of mutations shared between primary and metastatic lesions differed significantly between LDE and LIN cases (P = 0.923 and P = 0.928, respectively; Figure 4D–E). These findings indicate that the two evolutionary patterns are distinguished primarily by their patient‐specific phylogenetic topology rather than by the overall burden of shared mutations.

### 3.5 A shared SCAF4 truncation and transcriptomic effects of *SCAF4* depletion

**FIGURE 5:**
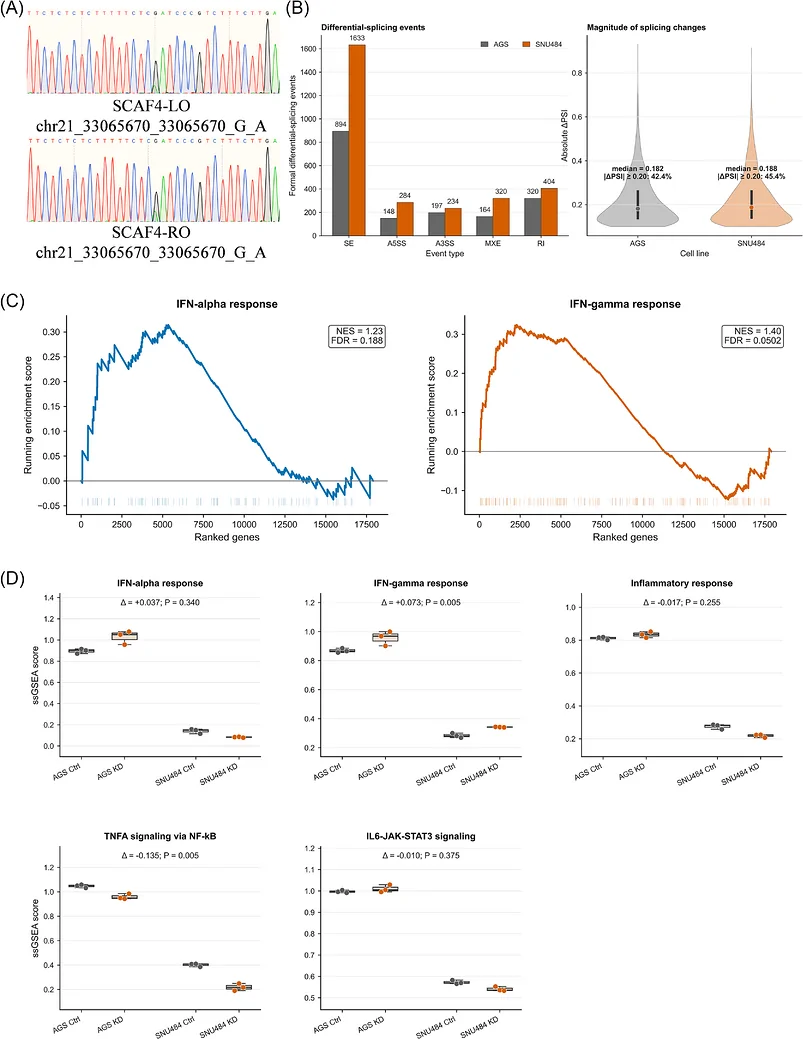
*SCAF4* loss is associated with differential splicing and selected interferon‐related transcriptional changes. (A) Sanger sequencing validating a somatic *SCAF4* mutation (G > A) in ovarian metastases. (B) rMATS differential‐splicing events in AGS and SNU484 cells (FDR ≤ 0.05, |ΔPSI| ≥ 0.10; *n* = 3 per group). Bars show event counts, and violin plots show |ΔPSI| distributions, medians and the proportions with |ΔPSI| ≥ 0.20. (C) Hallmark IFN‐response GSEA using genes ranked by the cell‐line‐adjusted edgeR statistic; NES and BH‐adjusted FDR are shown. (D) Sample‐level ssGSEA scores for five immune programs. Δ represents the stratified KD–control mean difference; *p*‐Values were calculated using 400 exact within‐cell‐line permutations with BH correction. PSI, per cent spliced in; SE, skipped exon; A5SS/A3SS, alternative 5′/3′ splice site; MXE, mutually exclusive exons; RI, retained intron; NES, normalised enrichment score; FDR, false discovery rate; KD, knockdown.

Among the candidate driver‐gene alterations, patient P05 harboured the same nonsense SCAF4 p.R484* alteration across the PRM, LN and bilateral OV specimens (Figure 2A). To validate this finding, we performed Sanger sequencing, which confirmed the presence of a G > A substitution at chromosome 21, position 33 065 670 (GRCh37/hg19) in the tumour tissue (Figure 5A). Immunohistochemical staining results demonstrated a loss of nuclear SCAF4 expression in ovarian metastatic lesions of patients with SCAF4 mutation (Figure S4A–C).

This specific mutation was consistently detected in both forward and reverse sequencing reads. Structural analysis revealed that this truncation maps specifically to the C‐terminal intrinsically disordered regions (IDRs) of *SCAF4*, domains predicted to drive biomolecular condensation and phase separation (Figure S5A,B). Consistent with its functional role as a splicing regulator, immunofluorescence staining validated that endogenous *SCAF4* exhibits predominant nuclear localisation in GC cells (Figure S5C).

We next examined whether *SCAF4* depletion was associated with altered RNA splicing in GC cells. RNA sequencing was performed in control and *SCAF4*‐knockdown AGS and SNU484 cells, with three biological replicates per group. rMATS analysis identified numerous differential alternative‐splicing events in both cell lines, encompassing skipped exons, alternative 5′ and 3′ splice sites, mutually exclusive exons, and retained introns (Figure 5B). Skipped‐exon events were the most frequent category, and a substantial proportion of the detected events showed marked changes in per cent spliced in (PSI), supporting an association between SCAF4 depletion and widespread alterations in alternative splicing.

Gene‐set enrichment analysis revealed positive enrichment of the Hallmark IFN‐α and IFN‐γ response programs following SCAF4 depletion, although neither met the prespecified FDR < 0.05 threshold (FDR = 0.188 and 0.0502, respectively; Figure 5C). In contrast, sample‐level ssGSEA identified a significant increase in the IFN‐γ response score and a decrease in TNF‐α signalling via NF‐κB after multiple‐testing correction, whereas the other immune‐related programs examined showed no significant changes (Figure 5D).

Together, these transcriptomic analyses indicate that SCAF4 depletion is associated with widespread alterations in RNA splicing and selective changes in immune‐related transcriptional programs, particularly IFN‐γ‐associated signalling.

### 3.6 *SCAF4* depletion is associated with enhanced proliferative, migratory and invasive phenotypes

**FIGURE 6:**
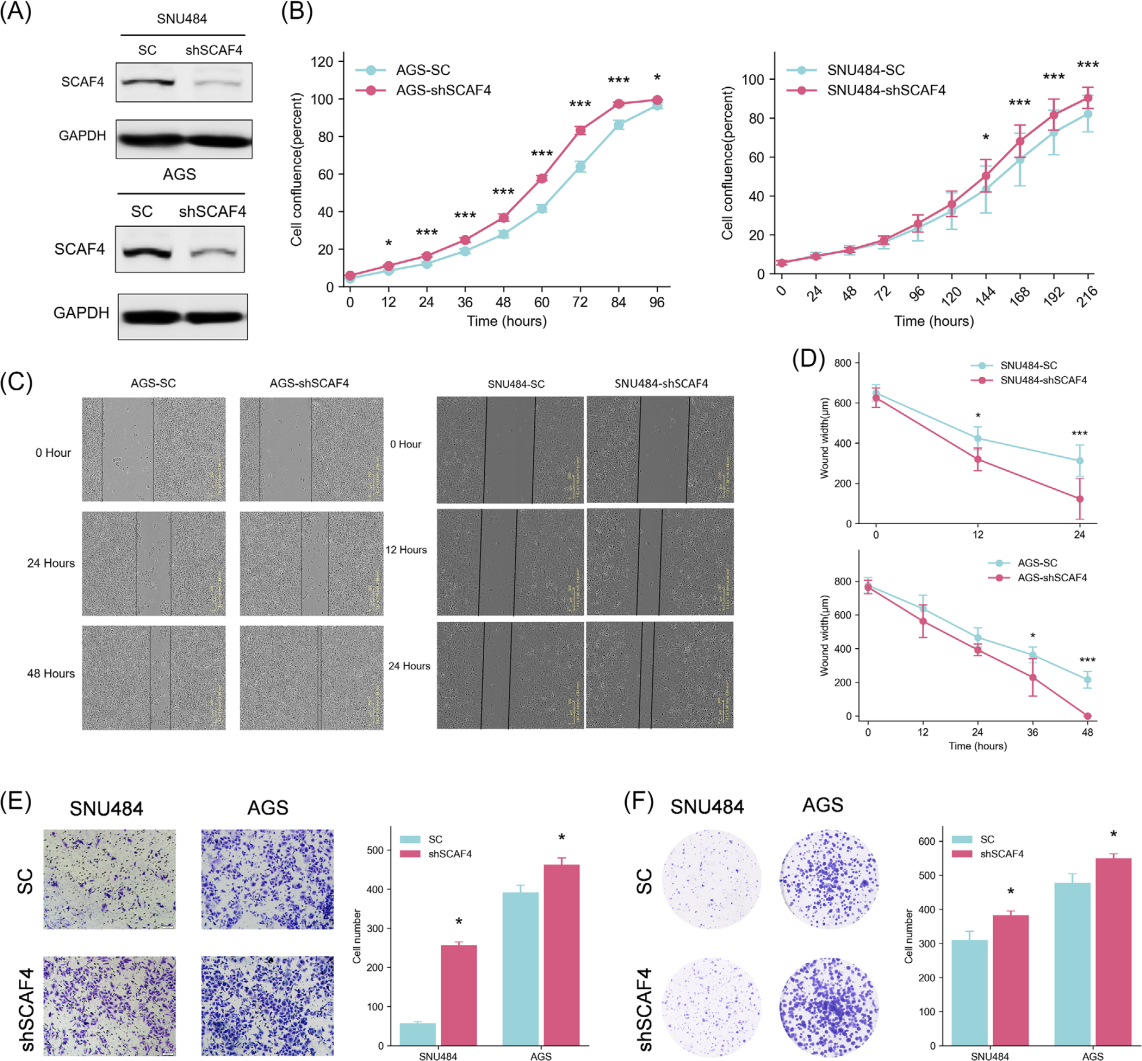
*SCAF4* depletion is associated with enhanced growth, migration, invasion, and clonogenicity in gastric cancer cells. (A) Western blot analysis of *SCAF4* expression in scramble control (SC) and *SCAF4*‐knockdown (*shSCAF4*) AGS and SNU484 cells. (B) Cell proliferation curves of SC and *shSCAF4* AGS and SNU484 cells. (C, D) Representative images and quantification of wound healing assays in SC and *shSCAF4* AGS and SNU484 cells. (E) Representative images and quantification of Transwell invasion assays in SC and *shSCAF4* AGS and SNU484 cells. (F) Representative images and quantification of colony‐formation assays in SC and *shSCAF4* AGS and SNU484 cells.

To examine the phenotypic consequences of *SCAF4* depletion, we established stable shRNA‐mediated *SCAF4* knockdown in two human GC cell lines, AGS and SNU484. Western blot analysis confirmed a significant and consistent reduction in *SCAF4* protein expression in the sh*SCAF4*‐transduced cells compared to scrambled control (SC) cells for both lines (Figure 6A).

We then assessed the impact of *SCAF4* loss on fundamental cancer cell behaviours. Notably, cell proliferation assays revealed that SCAF4 depletion was associated with increased cell growth/confluence in both cell lines (Figure 6B).

Consistent with these proliferative effects, SCAF4 depletion was also associated with enhanced cell motility. In wound healing assays, both AGS‐sh*SCAF4* and SNU484‐sh*SCAF4* cells exhibited a markedly accelerated closure of the wound gap compared to their respective control cells (Figure 6C,D). This heightened migratory capacity was further corroborated using Transwell invasion assays. We observed a significant increase in the number of invading cells that penetrated the Matrigel‐coated membrane in the *SCAF4*‐knockdown groups for both cell lines (Figure 6E). Furthermore, in colony formation assays, *SCAF4*‐depleted cells formed significantly more and larger colonies than control cells, indicating enhanced clonogenic growth (Figure 6F).

Collectively, SCAF4 depletion was associated with increased proliferative, wound‐closure, invasive, and clonogenic phenotypes in vitro.

## 4 DISCUSSION

For over a century, the route of GCOM has remained one of the most contentious issues in cancer metastasis research, with competing models including transcoelomic seeding and lymphatic spread, proposed but never definitively resolved.^16^, ^17^, ^18^ This long‐standing uncertainty hindered the development of effective therapies for this devastating disease. By integrating multi‐site whole‐exome sequencing with patient‐specific phylogenetic reconstruction, our study supports the existence of two major evolutionary patterns of OM: LDE and LIN. Rather than representing mutually exclusive universal mechanisms, these patterns provide a genomic framework for describing the heterogeneity of ovarian dissemination observed within our cohort.

Our findings substantially advance current knowledge of GCOM evolution beyond recent reports, which have primarily focused on static genomic comparisons between primary tumours and ovarian metastases. While several landmark studies have delineated the mutational landscape of GCOM, most rely on pairwise primary‐metastasis comparisons, treating metastasis as a binary endpoint and overlooking intermediate evolutionary steps and lymphatic involvement.^7^, ^17^, ^19^ For example, a recent study by Yu et al. categorised GCOM into parallel, linear, and intermediate evolutionary patterns based on primary–metastasis mutation sharing, but did not incorporate lymph node metastases into phylogenetic inference.^7^ Similarly, Lee et al. identified branched and diaspora‐like metastatic patterns in GC but did not stratify routes to the ovary or define pathway‐specific drivers.^17^ In contrast, our study incorporates matched lymph node metastases and multi‐site sampling, enabling patient‐level comparison of the phylogenetic relationships among primary, lymph node, ovarian, and peritoneal lesions. This multi‐site framework provides additional resolution for characterising the evolutionary heterogeneity of ovarian dissemination while avoiding inference of a specific physical metastatic route from phylogenetic topology alone.

Importantly, the LDE and LIN classifications were defined by the overall branching topology of the reconstructed phylogenies rather than by a simple difference in mutational burden. Neither the number nor the proportion of mutations shared between primary and metastatic lesions differed significantly between the two evolutionary groups. Thus, the distinction between LDE and LIN reflects differences in the phylogenetic relationships among anatomical sites rather than quantitatively greater mutation sharing in one group. The LDE topology is consistent with an evolutionary relationship involving lymph node‐associated metastatic lineages, whereas LIN is compatible with ovarian dissemination occurring independently of the sampled lymph node lineage. However, phylogenetic reconstruction infers ancestral relationships among tumour samples and does not directly observe the physical route or direction of tumour‐cell dissemination. Accordingly, LIN should not be interpreted as definitive evidence of a specific transcoelomic or hematogenous route.

Beyond DNA‐level alterations, dysregulation of RNA regulatory processes has increasingly been implicated in GC progression and metastasis.^20^ Within this framework, *SCAF4* emerged as an understudied candidate metastasis‐associated factor. In patient P05, the same nonsense alteration, *SCAF4* p.Arg484Ter (p.R484*), was detected across the primary tumour and multiple metastatic lesions, consistent with an early shared event. *SCAF4* was included among the 299 pan‐cancer driver genes identified by Bailey et al. using a consensus computational approach,^8^ but it has not been established as a canonical driver in GC. The predicted truncation removes a substantial portion of the C‐terminal sequence, including predicted IDRs. Because IDRs frequently contribute to dynamic protein–protein and protein–RNA interactions and can participate in biomolecular condensation,^20^, ^21^, ^22^ their loss provides a plausible structural basis for altered SCAF4 function. Nevertheless, our study did not directly assess SCAF4 phase‐separation behaviour, and the proposed relationship between truncation, IDR loss, and altered biomolecular condensation remains speculative.

Functional and transcriptomic analyses provided additional support for investigating loss of SCAF4 function. SCAF4 depletion in AGS and SNU484 GC cells was associated with increased proliferative, migratory, invasive, and clonogenic phenotypes in vitro. RNA sequencing further showed widespread changes in alternative splicing following SCAF4 depletion. Interferon‐related transcriptional programs also showed selective alterations: Hallmark IFN‐α and IFN‐γ response gene sets showed positive enrichment in GSEA, although neither met the prespecified FDR < 0.05 threshold, whereas sample‐level ssGSEA identified an increase in the IFN‐γ response score after multiple‐testing correction. These findings suggest that SCAF4 loss may influence both RNA processing and selected immune‐related transcriptional programs. However, they do not establish that the interferon‐related changes are directly caused by particular SCAF4‐dependent splicing events. In addition, the cell‐line experiments modelled SCAF4 depletion rather than the patient‐specific p.R484* truncation. The potential SCAF4 loss–splicing–interferon relationship should therefore be regarded as a working hypothesis rather than an established mechanistic pathway. These data are also insufficient to establish SCAF4 status as a biomarker of immune checkpoint inhibitor response.^23^ Future studies integrating SCAF4 status with established immune biomarkers, tumour‐immune microenvironment profiling, and clinical treatment‐response data will be required to evaluate its potential immunotherapy relevance.

Several limitations should be acknowledged. First, despite the extensive multi‐site sampling, the cohort included only 17 patients, which limits the statistical power to identify recurrent metastasis‐associated alterations and the generalizability of the proposed evolutionary framework. Validation in larger independent cohorts with systematic sampling of primary, lymph node, ovarian, and peritoneal lesions will therefore be important. Second, treatment exposure varied across patients and sampling time points and may have influenced mutation accumulation, mutational signatures, and clonal selection.^24^, ^25^ Accordingly, the reconstructed phylogenies should be interpreted as reflecting tumour evolution over each patient's observed clinical course, including potential treatment‐mediated selection, rather than untreated natural evolution. Third, quantitative pathology‐based post‐microdissection tumour‐cell percentages were not systematically recorded for these archival specimens. Although tumour‐rich regions were enriched by pathology‐guided laser microdissection, residual inter‐sample variation in tumour cellularity may have affected somatic variant detection and phylogenetic inference. The Bayesian framework implemented in Treeomics was used to mitigate the influence of sequencing uncertainty and variable tumour cellularity, although these effects cannot be completely eliminated. Finally, the functional analyses were based on *SCAF4* depletion rather than mutation‐specific models. Additional rescue, mutation‐specific, and in vivo studies, together with validation of individual differential‐splicing events, will be needed to define the mechanistic consequences of *SCAF4* alteration more precisely. Accordingly, *SCAF4* should be regarded as a candidate metastasis‐associated factor rather than an established metastatic driver.

In conclusion, multi‐site genomic profiling and phylogenetic reconstruction support two major evolutionary patterns of OM in GC, characterised by lymph node‐dependent and lymph node‐independent relationships. These findings highlight the evolutionary heterogeneity of GCOM and provide a patient‐level framework for investigating its metastatic biology. The identification of *SCAF4* as a candidate metastasis‐associated factor further illustrates how early shared genomic alterations may contribute to metastatic phenotypes and provides a basis for future mechanistic investigation.

## AUTHOR CONTRIBUTIONS

Peiyu Zhu, Xiaofang Xing, Ke Ji and Lingqian Wang contributed equally to this work. Biao Fan, Zhaode Bu and Jiafu Ji conceived the study, designed and supervised the project. Peiyu Zhu, Xiaofang Xing, Ke Ji and Lingqian Wang performed the majority of the experiments and analysed the data. Peiyu Zhu, Xiaofang Xing and Biao Fan drafted the manuscript. Zhongwu Li, Shuqin Jia, Longtao Huangfu and Xiaomei Li provided technical assistance and contributed to data acquisition. All authors reviewed, discussed, and approved the final version of the manuscript.

## CONFLICT OF INTEREST STATEMENT

The authors declare no conflict of interest.

## ETHICS STATEMENT

The study of human tumour samples was performed according to the Declaration of Helsinki and Good Clinical Practice and approved by the Ethical Committee of Beijing Cancer Hospital (2024YJZ56).

## PATIENT CONSENT STATEMENT

Informed written consent was obtained from all participants.

## Supporting information



Supporting Information

Supporting Information

Supporting Information

Supporting Information

## Data Availability

The raw sequencing data generated in this study have been deposited in the National Genomics Data Centre (NGDC), China National Centre for Bioinformation, under BioProject accession PRJCA067053. The whole‐exome sequencing data are deposited under HRA accession HRA020118, and the RNA‐sequencing data from SCAF4‐depleted AGS and SNU484 gastric cancer cells are deposited under HRA accession HRA020395.
