# Supplementary material for: Multi‐site sequencing supports dual‐track evolution and identifies candidate metastasis‐associated factors in gastric cancer ovarian metastasis
Source: Clin Transl Med. 2026 Sep 21;16(9):e70847. doi: 10.1002/ctm2.70847 (PMC13591221; doi:10.1002/ctm2.70847)
Supplement: Supplementary file 1 [file CTM2-16-e70847-s003.pdf]

Figure S1

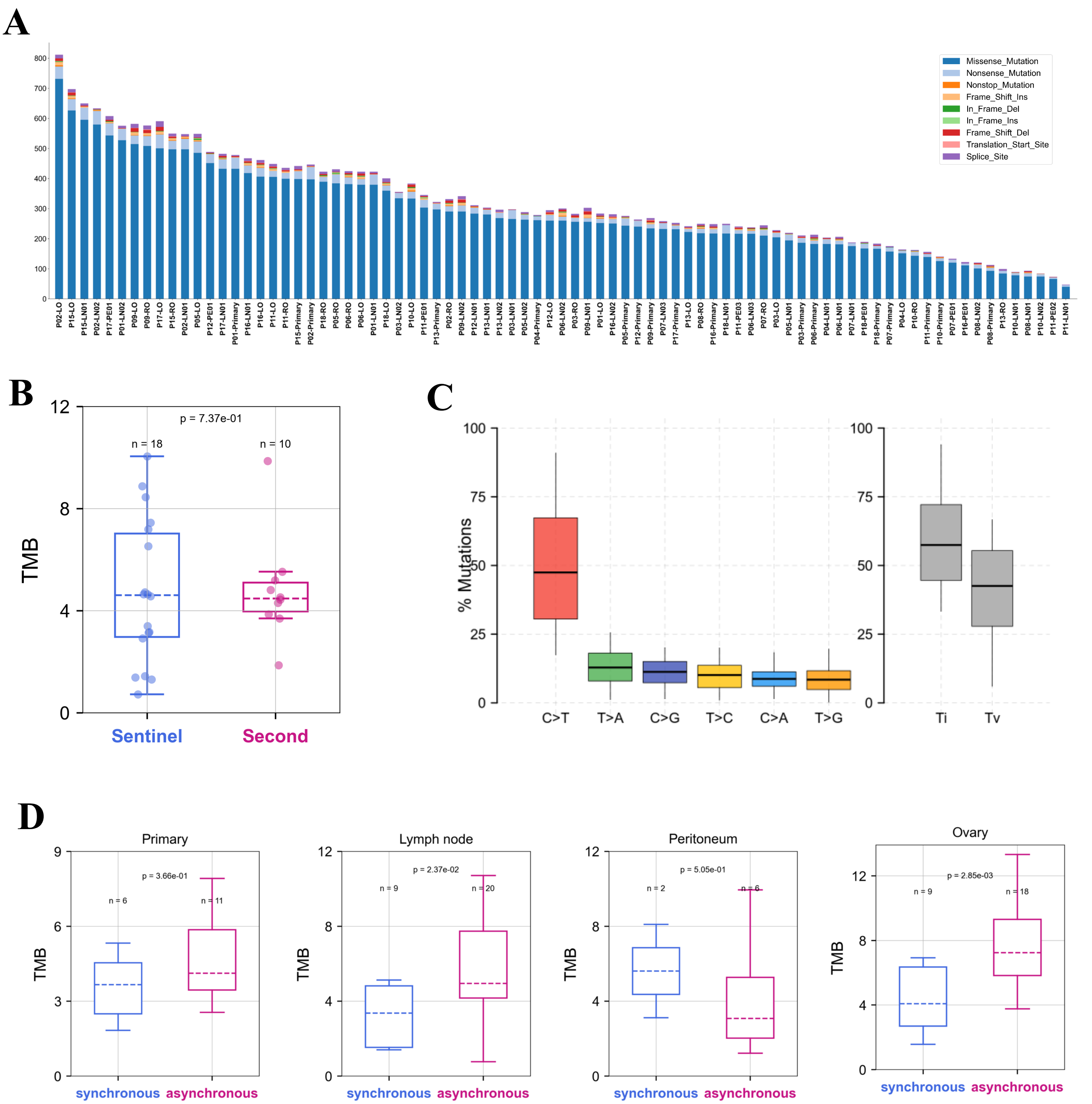

Supplementary Figure 1. Spatio-temporal mutational landscape.

A Mutational spectrum. Distribution of somatic variant types across the 81-sample cohort. **B** Sentinel vs. Secondary. TMB comparison showing no significant difference between early and late metastatic sites (p=0.737). **C** Base substitutions. Global dominance of C>T transitions and Ti/Tv ratios. **D** Timing-dependent TMB. TMB comparison between synchronous and asynchronous samples across anatomical sites. Note significantly elevated TMB in asynchronous lymph node (p=0.0237) and ovarian ($p=2.85\times10^{-3}$) metastases**.**

Figure S2

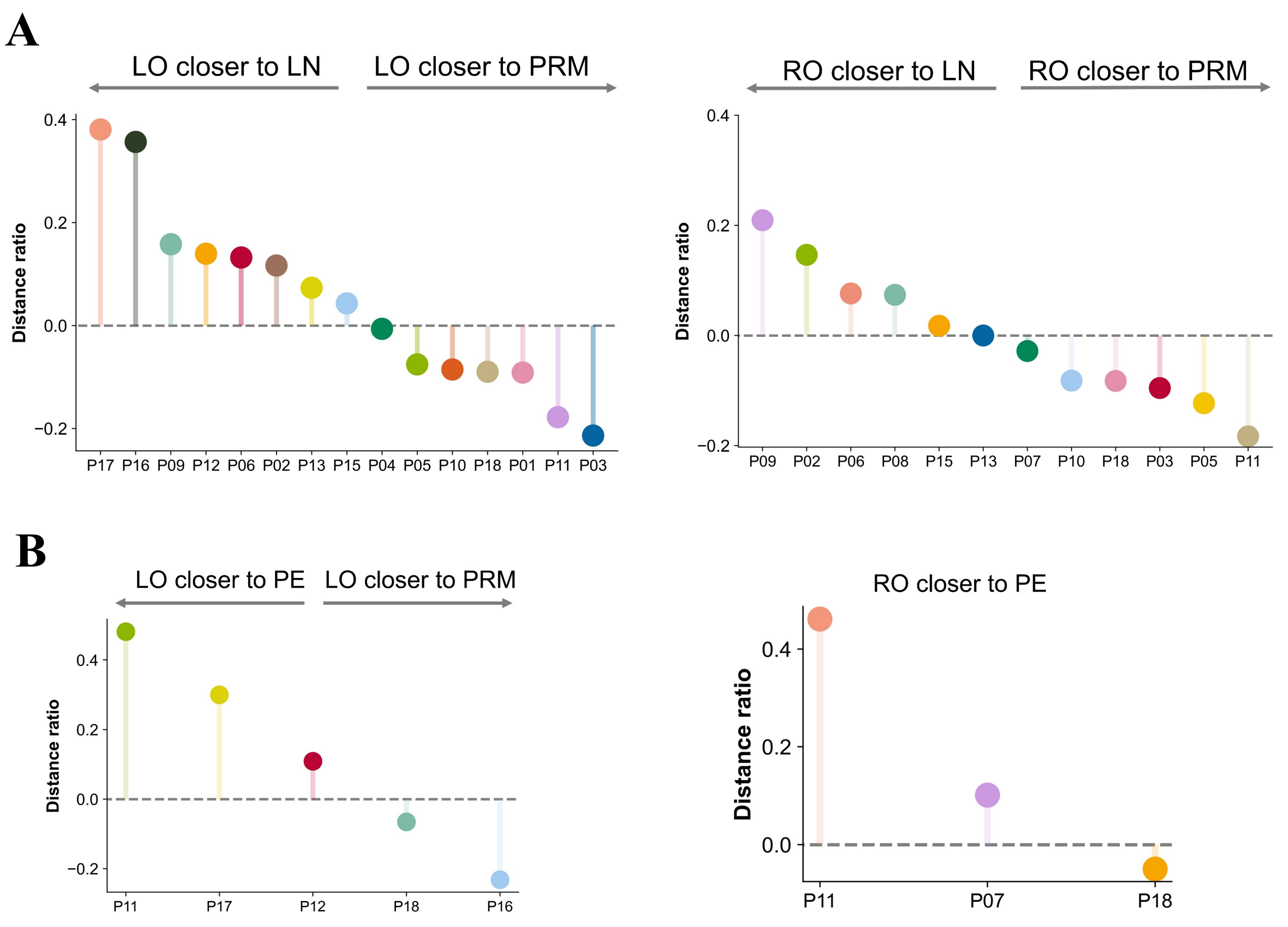

Supplementary Figure 2. Evolutionary distance quantification of GCOM.

A,B Genetic distance between primary and metastatic foci.

Figure S3

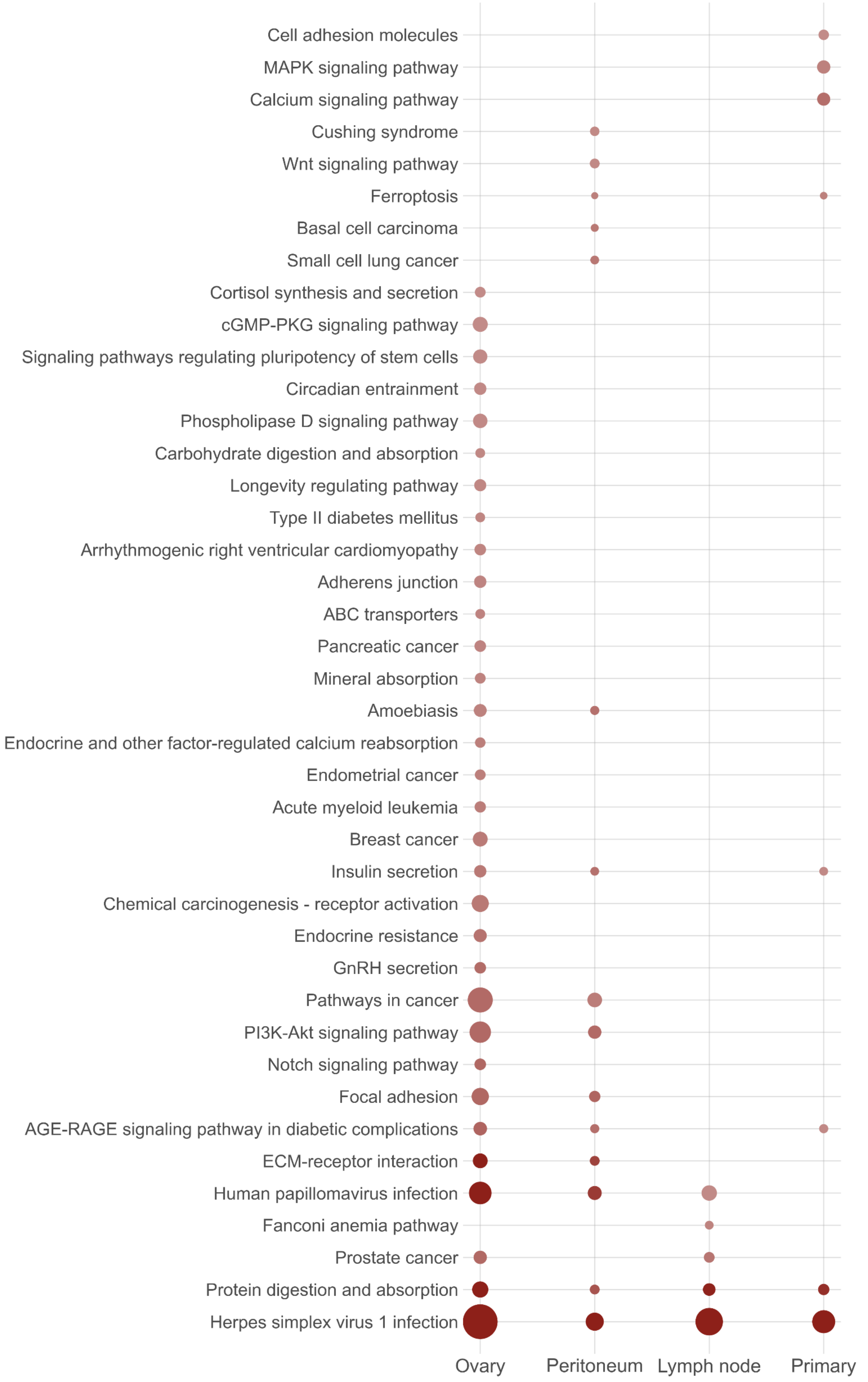

Supplementary Figure 3. GO pathway enrichment of primary and metastatic foci.

Figure S4

A B C

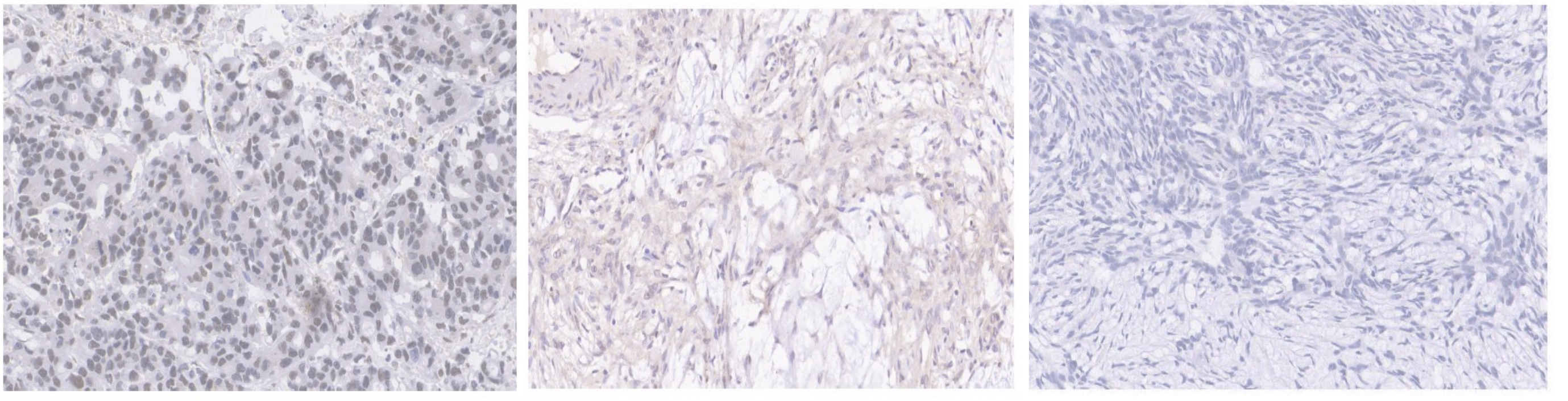

Supplementary Figure 4. Loss of nuclear SCAF expression in SCAF-mutated gastric cancer samples.

A Primary gastric lesion in patients without SCAF4 mutation. B Left-ovarian lesion in patients with SCAF4 mutation. C Right-ovarian lesion in patients with SCAF4 mutation.

Figure S5

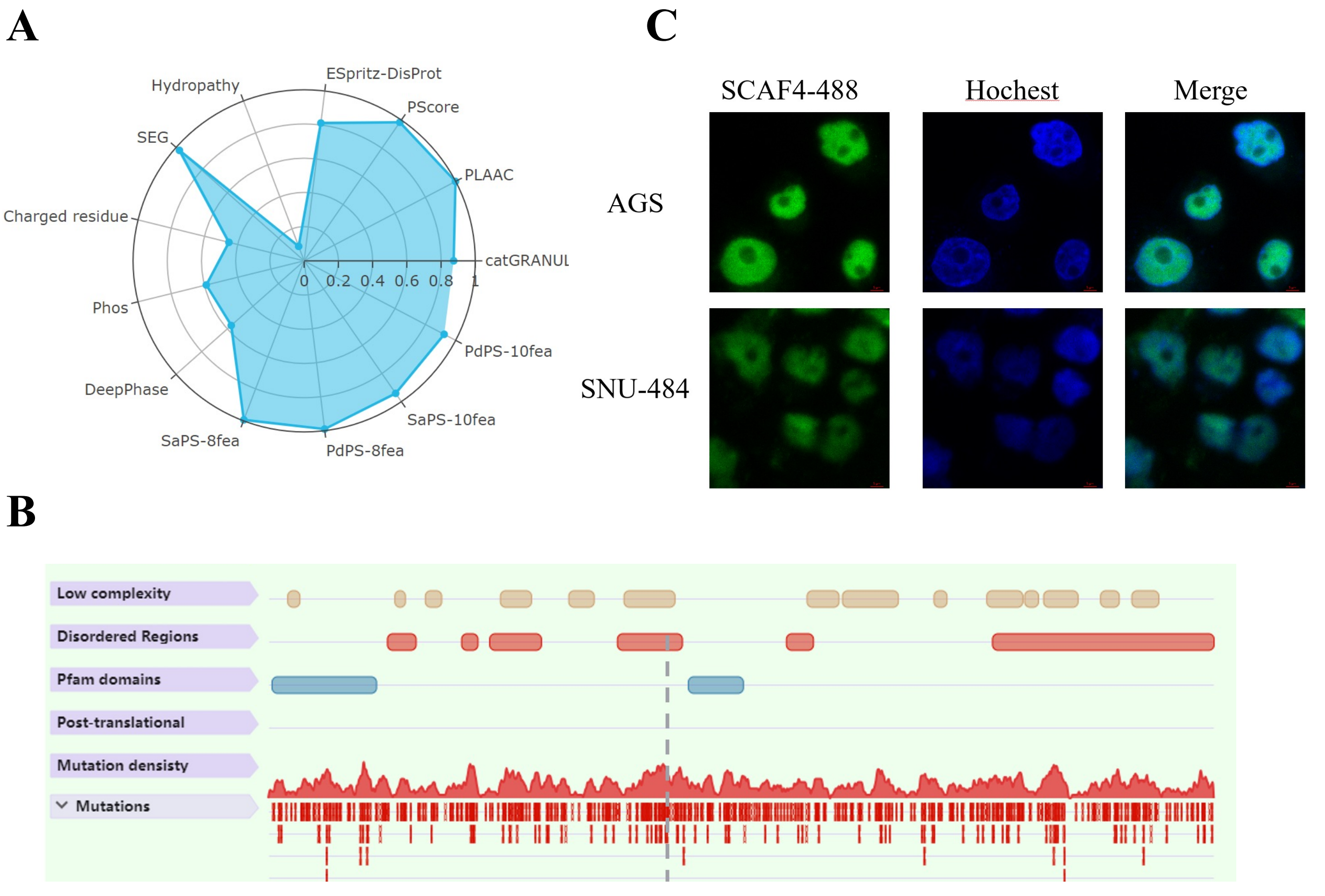

Supplementary Figure 5. Structural characterization and nuclear localization of *SCAF4*.

A Radar chart illustrating the multidimensional computational prediction of *SCAF4* phase separation potential. Scores from various algorithms (including catGRANUL, PLAAC, and DeepPhase) indicate a high propensity for liquid-liquid phase separation (LLPS), as shown by the extensive coverage in the outer rings (high probability scores). **B** Domain architecture and mutation landscape of *SCAF4*. The tracks display predicted intrinsically disordered regions (IDRs, red blocks), Pfam domains (blue blocks), and low-complexity regions (beige). The bottom track illustrates mutation density (red peaks) and individual mutation sites (vertical ticks) from the cohort. The vertical dashed line highlights the approximate position of the identified truncating mutations, demonstrating the loss of C-terminal IDRs. **C** Immunofluorescence analysis of endogenous *SCAF4* subcellular distribution in gastric cancer cell lines (AGS and SNU-484). *SCAF4* (green) exhibits predominant nuclear localization, consistent with its functional role as a splicing regulator. Nuclei were counterstained with Hoechst (blue).
